# *Passiflora edulis* Seed Oil in an Experimental Zebrafish Model of Hyperglycemia During Embryogenesis: Evaluation of Free Glucose Modulation, Oxidative Stress, Antioxidant Gene Expression, and Developmental Safety

**DOI:** 10.3390/molecules31162927

**Published:** 2026-08-21

**Authors:** Elaine L. S. S. Mendonça, Felipe C. Silva, José W. A. C. Silva, Rafael L. B. Oliveira, Igor F. P. Silva, Henrique F. Goulart, Antônio E. G. Santana, João I. Soletti, Alane C. M. Oliveira, Marília O. F. Goulart, Jadriane A. Xavier

**Affiliations:** 1 National Institute of Science and Technology in Bioanalytics Lauro Kubota (INCTBio-LK), University of Campinas, Campinas 13083-861, SP, Brazil; elaine.mendonca@fanut.ufal.br; 2 Institute of Chemistry and Biotechnology, Federal University of Alagoas, Maceió 57072-900, AL, Brazil; felipe.silva@iqb.ufal.br (F.C.S.); jose.walber@iqb.ufal.br (J.W.A.C.S.); rafael.oliveira@iqb.ufal.br (R.L.B.O.); igor.pereira@iqb.ufal.br (I.F.P.S.); 3 Agricultural Engineering and Sciences Campus, Federal University of Alagoas, Rio Largo 57100-000, AL, Brazil; henrique.goulart@ceca.ufal.br (H.F.G.); aegs@ceca.ufal.br (A.E.G.S.); 4 Center of Technology, Federal University of Alagoas, Maceió 57072-900, AL, Brazil; jsoletti@ctec.ufal.br; 5 College of Nutrition, Federal University of Alagoas, Maceió 57072-900, AL, Brazil; alanecabral@gmail.com

**Keywords:** biotechnology, circular economy, *Danio rerio*, embryotoxicity, *nfe2l2a*, ROS

## Abstract

*Passiflora edulis* seed oil (PESO) is a rich source of lipophilic bioactive compounds with potential to modulate oxidative stress and free glucose levels. This preclinical study aimed to evaluate the toxicological profile, antioxidant and glucose-modulating effects of PESO using hyperglycemic *Danio rerio* embryos (AB strain). PESO was chemically characterized by GC-FID/GC-MS after derivatization to fatty acid methyl esters. In animal studies, the median lethal concentration (LC_50_) of PESO at 96 h post-exposure (hpe) was determined using concentrations ranging from 12.5 to 400 μg/mL, and sublethal concentrations corresponding to 1/10 and 1/20 of the LC_50_ were selected for further investigations, including survival rate up to 144 hpe, continuous and pulsatile hyperglycemia assays (total free glucose was quantified in washed embryo or larval homogenates using an Accu-Chek Active^®^ glucometer) (Roche Diabetes Care GmbH, Mannheim, Germany), total reactive oxygen species (ROS) production, and antioxidant gene expression at 96 hpe. Data were analyzed by nonlinear regression or analysis of variance followed by appropriate post hoc tests, with significance set at *p* ≤ 0.05. Chemical characterization identified linoleic acid (C18:2) as the predominant fatty acid. PESO exhibited an LC_50_ of 191.1 µg/mL. PESO at 9.5 and 19.1 µg/mL achieved embryonic survival above 85% and reduced total free glucose values in washed embryo or larval homogenates under pulsatile and continuous hyperglycemic conditions (*p* ≤ 0.05), although less effective than insulin (0.1 U/mL). PESO attenuated ROS production by 2% (*w*/*v*) glucose (*p* ≤ 0.05). PESO treatment was associated with distinct concentration-related expression profiles among the selected antioxidant-related genes: *sod1* and *cat* expression increased at both concentrations, *sod2*, *gpx1a*, and *gsr* increased at 19.1 µg/mL, and *nfe2l2a* increased at 9.5 µg/mL (*p* ≤ 0.05). At the selected sublethal concentrations, PESO showed low embryotoxicity and was associated with reduced total free glucose values, partial attenuation of ROS accumulation, and changes in selected antioxidant-related transcripts in embryos exposed to hyperglycemic conditions.

## 1. Introduction

Traditional medicine highlights vegetable oils for their emollient properties and as vehicles for bioactive compounds [1,2]. Although historically associated with topical use, vegetable oils have attracted increasing scientific and commercial interest because fatty acids and minor constituents, including tocopherols, phytosterols, and polyphenols, have been reported in these lipid matrices and associated with antioxidant and metabolic properties [3]. These biological effects may be associated with the extraction methods used, as they play central roles in preserving chemical composition and ensuring physiological safety, with cold pressing being particularly effective at mitigating thermal degradation and solvent contamination [4].

Among vegetable oils, *Passiflora edulis* seed oil (PESO) stands out for its abundant seed availability, driven by its wide geographic distribution and near-continuous fruit production [5]. However, PESO remains largely unexplored, largely due to technological challenges related to its incorporation into aqueous systems and the scarcity of robust toxicological studies [4,6]. Toxicological assessment is particularly relevant because substances of natural origin are not inherently safe and may exhibit adverse effects, including carcinogenic, mutagenic, and teratogenic activities, as well as specific physiological effects, such as emmenagogue action, when not adequately investigated [7,8].

In this context, the zebrafish (*Danio rerio*) has been established as a highly efficient alternative model for toxicology, pharmacology, and metabolic studies. Notably, the zebrafish exhibits key features relevant to embryonic studies: the transparency of its embryos allows real-time observation of embryogenesis, and its genetic proximity to humans (with approximately 70% gene homology and orthologs for 84% of human disease-associated genes), combined with its easy handling, low maintenance cost, and high fecundity, make it an ideal model for experimental investigations of chemicals and pathophysiological processes [9,10].

Among the latter, hyperglycemia has been recognized as a key factor in systemic inflammation, increased ROS production, and structural damage to sensitive tissues [11]. In human pregnancy, maternal hyperglycemia can affect fetal growth, alter insulin secretion mechanisms, and increase the risk of chronic diseases later in life through fetal programming [11,12]. Thus, hyperglycemic embryonic models offer a unique opportunity to simultaneously evaluate the safety and bioactivity of natural compounds [13,14,15]. The present study aimed to establish the toxicological profile and evaluate the effects of PESO on total free glucose, ROS accumulation, selected antioxidant-related transcripts, and developmental safety in zebrafish embryos exposed to experimentally induced hyperglycemia.

## 2. Results

### 2.1. Fatty Acid Composition of PESO

The fatty acid composition of PESO was determined after derivatization to fatty acid methyl esters (FAMEs). The four principal compounds were assigned to their corresponding fatty acids: methyl palmitate, methyl linoleate, methyl cis-9-octadecenoate, and methyl octadecanoate (Table 1).

Linoleic acid (C18:2) was the predominant fatty acid, representing 66.93% of the total chromatographic peak area. Oleic acid (C18:1 cis-9) was the second most abundant fatty acid, accounting for 17.03%, followed by palmitic acid (C16:0; 11.24%) and stearic acid (C18:0; 4.80%). Overall, unsaturated fatty acids accounted for 83.96% of the detected FAMEs, whereas saturated fatty acids represented 16.04%. This profile confirms the predominance of unsaturated fatty acids in PESO, particularly linoleic acid.

### 2.2. Embryotoxicity, Median Lethal Concentration (LC_50_) Determination, and Survival Analysis

Physicochemical parameters (temperature and pH values) of the embryo medium were monitored throughout the 96 h post-exposure (hpe) period to ensure stable experimental conditions. The medium remained thermally stable, with a mean temperature of 26.1 ± 0.1 °C, and exhibited a nearly neutral pH (6.9 ± 0.1), with no significant fluctuations across PESO concentrations. No effects were observed in the embryos.

**Figure 1:**
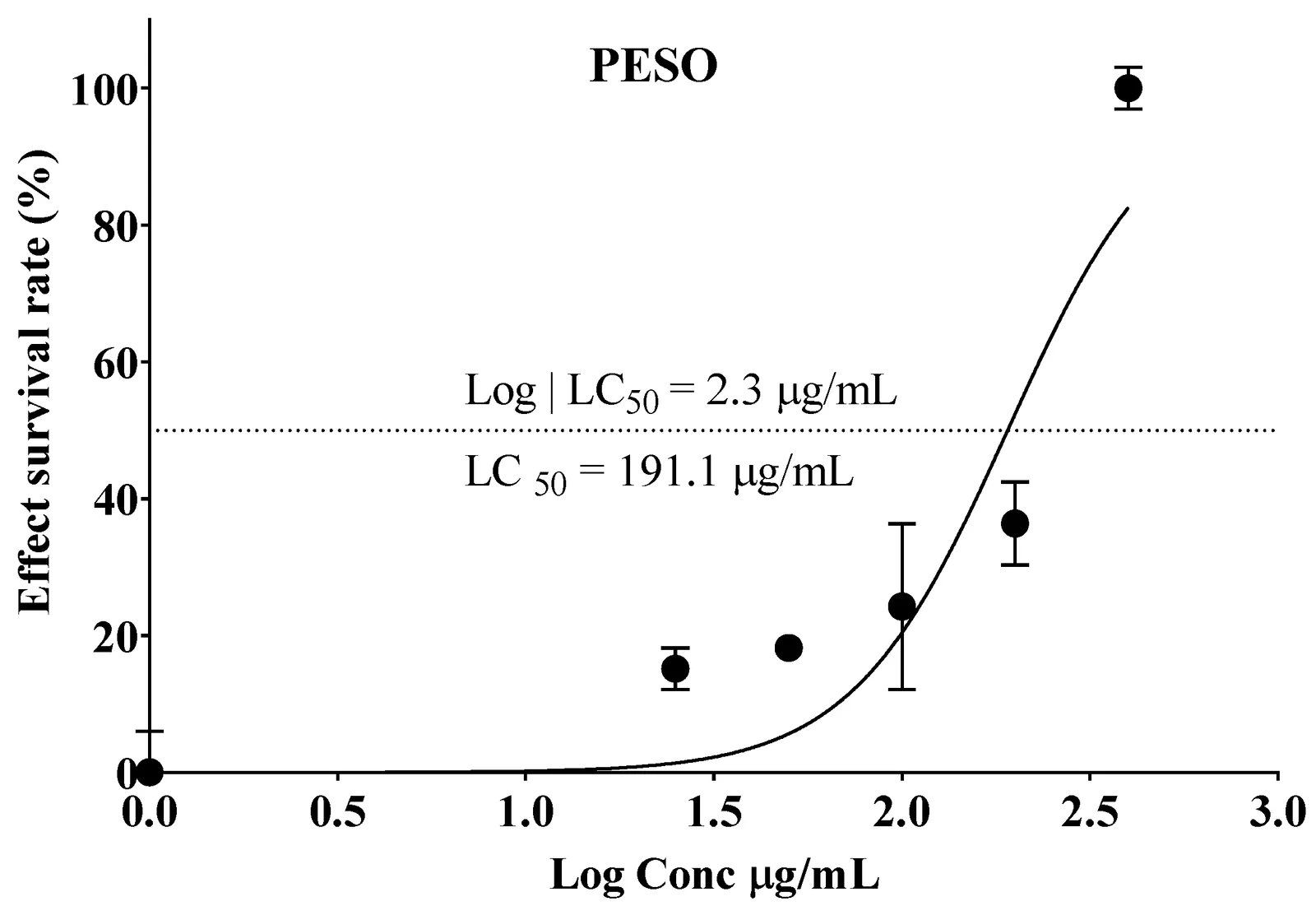
Concentration–response curves illustrating the effect of *Passiflora edulis* seed oil (PESO) on the survival of *Danio rerio* embryos after 96 h of exposure. Data are expressed as mean ± SEM from three independent biological experiments, each including 20 individually exposed embryos per PESO concentration, corresponding to a total of 60 embryos per concentration. LC_50_ values were calculated by nonlinear regression using the log(inhibitor) versus normalized response model with a variable slope in GraphPad Prism 9 (GraphPad Software, San Diego, CA, USA). The estimated LC_50_ for PESO was 191.1 µg/mL (95% CI: 123.5–269.0 µg/mL; logLC_50_ = 2.3).

Embryo mortality increased in a concentration-dependent manner, particularly at 200 and 400 μg/mL, which also showed the highest incidence of developmental malformations. The concentration–response relationship was further characterized using nonlinear regression analysis (Figure 1), yielding an estimated median lethal concentration (LC_50_) of 191.1 µg/mL (95% CI: 123.5–269.0 µg/mL; logLC_50_ = 2.3).

**Figure 2:**
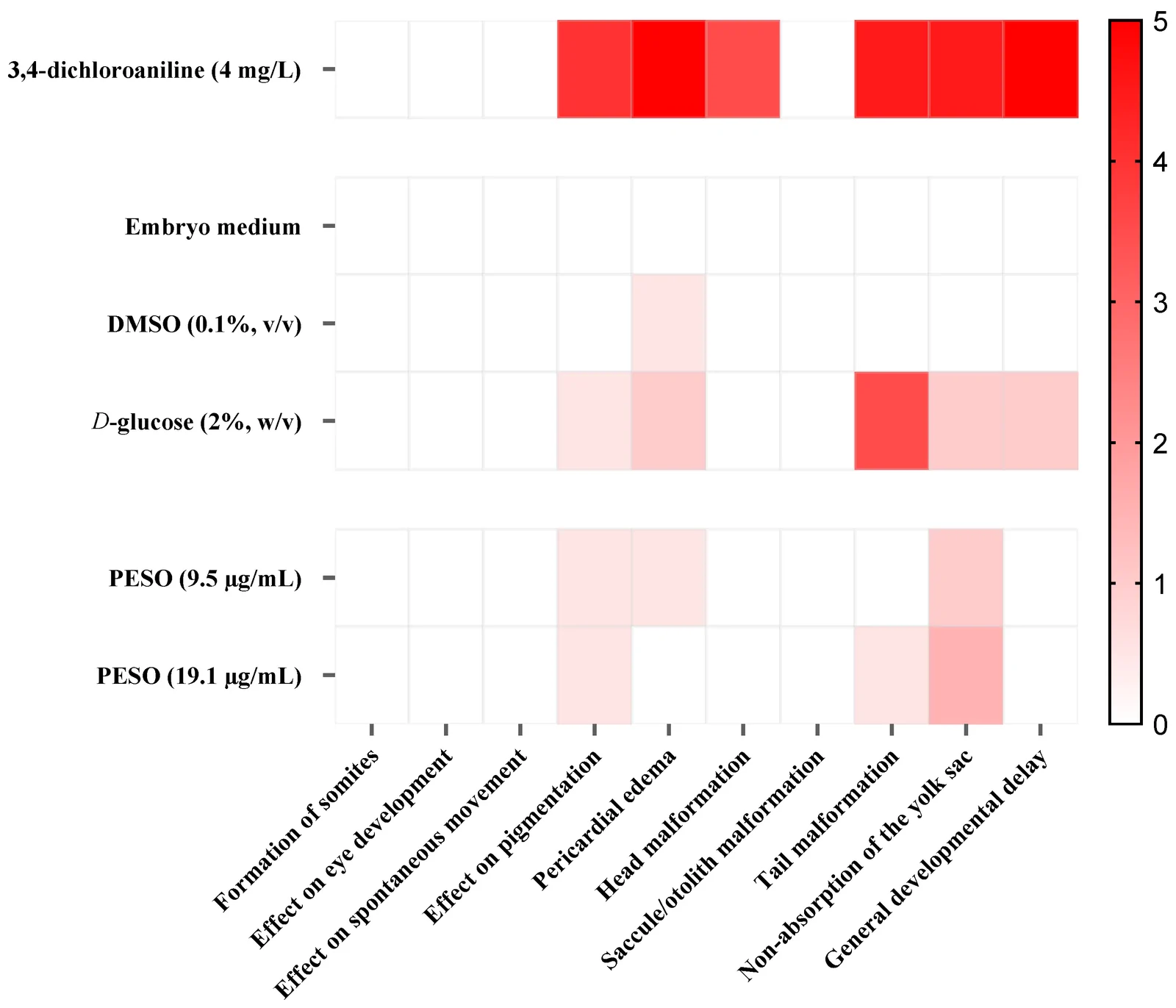
Heatmap of developmental abnormalities in *Danio rerio* embryos exposed to *Passiflora edulis* seed oil (PESO). Evaluated endpoints included somite formation, eye development, spontaneous movement, pigmentation, pericardial edema, yolk sac malformation, tail malformation, non-absorption of the yolk sac, and general developmental delay. Positive control (3,4-dichloroaniline), negative control (embryo medium), solvent control (0.1% dimethylsulfoxide—DMSO), and *D*-glucose (2%, hyperglycemia control) groups are shown. Data are expressed as mean ± SEM (*n* = 3). Tested PESO concentrations were 19.1 µg/mL (1/10 LC_50_) and 9.5 µg/mL (1/20 LC_50_). Color intensity represents the descriptive severity score assigned to each developmental endpoint, ranging from 0 (no observable alteration; white) to 5 (highest observed severity; red). The heatmap is presented as a semiquantitative descriptive visualization.

Based on the LC_50_ value determined for PESO, two sublethal concentrations (1/10 and 1/20 of the LC_50_; 19.1 and 9.5 μg/mL, respectively) were selected for subsequent embryotoxicity assessment. Developmental endpoints were systematically evaluated at 96 hpe to characterize potential morphological alterations associated with PESO exposure (Figure 2).

The positive control (3,4-dichloroaniline) led to the highest frequency and diversity of abnormalities, including alterations in pigmentation, pronounced pericardial edema, tail and head malformations, impaired yolk sac absorption, and marked general developmental delay, confirming the assay’s sensitivity. As expected, embryos from the negative control and solvent control groups exhibited normal development, with only one isolated case of pericardial edema in the solvent control group (Figure 2).

Embryos exposed to 2% (*w*/*v*) *D*-glucose presented moderate developmental disturbances, particularly increased pigmentation defects and tail malformations. Notably, these alterations partially overlapped with those observed in the positive-control experiment, though they occurred at lower frequency and intensity.

The PESO exposure at concentrations of 9.5 µg/mL resulted in a low incidence of abnormalities, primarily mild pigmentation changes, discrete pericardial edema, and occasional yolk sac non-absorption. For PESO (at 19.1 µg/mL), the frequency of alterations remained low but slightly higher than shown above, with isolated occurrences of tail malformation, pigmentation defects, and mild yolk sac non-absorption. Overall, both PESO sublethal concentrations produced only subtle morphological effects, with no indication of severe structural abnormalities (Figure 2).

**Figure 3:**
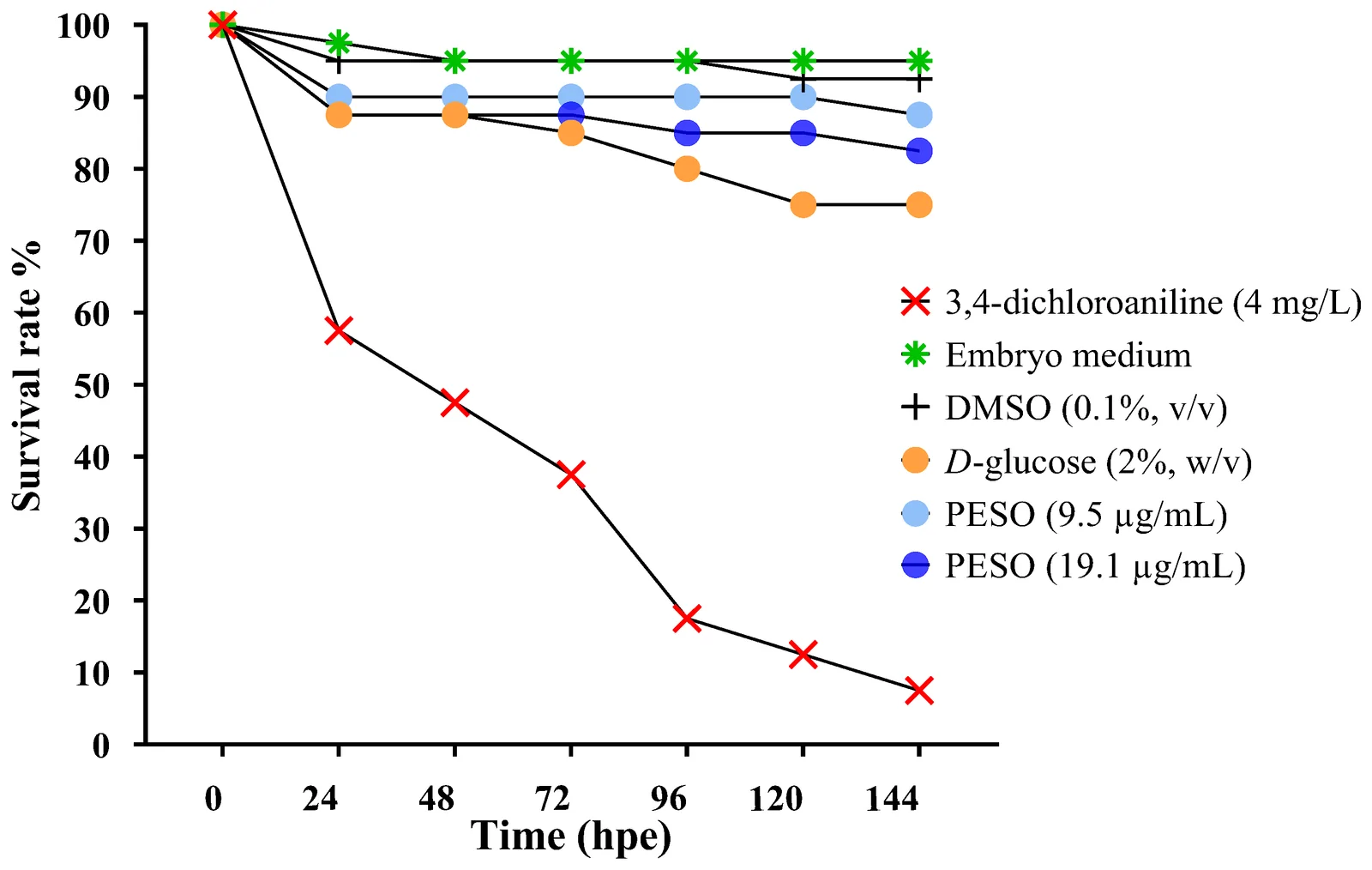
Survival rates of *Danio rerio* embryos exposed to 1/10 and 1/20 of the LC_50_ concentrations of *Passiflora edulis* seed oil (PESO) over 144 h. Positive control (3,4-dichloroaniline), negative control (embryo medium), solvent control (0.1% DMSO), and *D*-glucose (2%, hyperglycemia control) groups are shown. Data are expressed as mean ± SEM (*n* = 3). Tested PESO concentrations were 19.1 µg/mL (1/10 LC_50_) and 9.5 µg/mL (1/20 LC_50_). Survival was monitored at 0, 24, 48, 72, 96, and 144 hpe.

To further characterize potential sublethal effects of PESO, embryo survival was monitored for up to 144 hpe at the two selected concentrations (9.5 and 19.1 μg/mL) and compared with the positive control. As expected, the positive control (3,4-dichloroaniline) led to a marked and progressive decline in survival, dropping to less than 10% at 144 hpe, confirming assay responsiveness (Figure 3). Embryos from the negative and solvent controls maintained consistently high survival rates (>90%) throughout the experiment.

Exposure to 2% (*w*/*v*) *D*-glucose resulted in a moderate reduction in survival after 96 hpe, decreasing to 75% at 144 hpe, which is consistent with the known impact of hyperglycemia on embryo viability.

At both PESO concentrations, embryo survival remained stable throughout the exposure period. Embryos treated with 9.5 μg/mL exhibited survival rates comparable to those of the control groups, with no significant decline over time. Similarly, embryos exposed to 19.1 μg/mL exhibited survival above 85% up to 144 hpe, indicating that these sublethal concentrations did not compromise viability. Overall, PESO at 1/10 and 1/20 of the LC_50_ did not induce time-dependent lethality, supporting the selection of these concentrations for subsequent functional assays (Figure 3).

### 2.3. Effects of PESO on Total Free Glucose in Washed Embryo and Larval Homogenates Under Hyperglycemic Conditions

**Figure 4:**
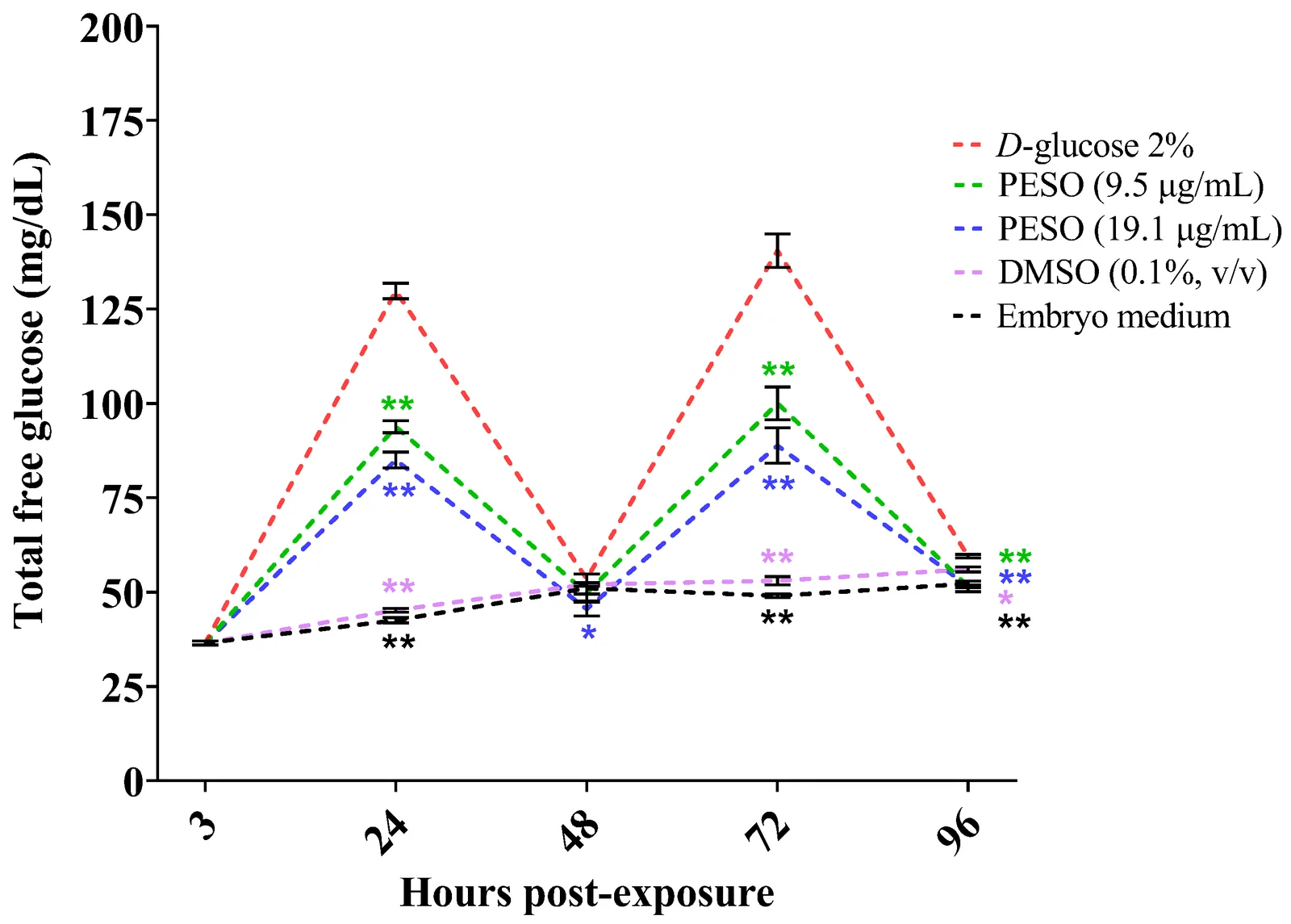
Effect of *Passiflora edulis* seed oil (PESO) on total free glucose measured in washed embryo or larval homogenates under a pulsatile hyperglycemia protocol in *Danio rerio*. The pulsatile protocol was designed to evaluate embryo responses during alternating glucose-exposure and glucose-free recovery periods without pharmacological intervention; therefore, no insulin-treated comparison was included. Embryos were subjected to alternating periods of exposure to 2% D-glucose and glucose-free recovery intervals throughout development: 3–24 hpe (hyperglycemia), 24–48 hpe (normoglycemia), 48–72 hpe (hyperglycemia), and 72–96 hpe (normoglycemia). PESO (9.5 and 19.1 µg/mL) was administered during the respective exposure periods. Total free glucose was measured in washed embryo or larval homogenates at 3, 24, 48, 72, and 96 hpe using a portable glucometer. Statistical analysis was performed using two-way ANOVA followed by Dunnett’s post hoc test to compare each treatment group with the hyperglycemic control (2% *D*-glucose). Significance is indicated as * *p* < 0.05 and ** *p* < 0.001. Symbols were color-coded by treatment groups for clarity. Data are expressed as mean ± SEM (*n* = 3).

Under the pulsatile hyperglycemia protocol, zebrafish embryos were alternately exposed to glucose-containing medium (2% *D*-glucose) and embryo medium without glucose at defined developmental time points (3, 24, 48, 72, and 96 hpe) (Figure 4).

At 24, 72, and 96 hpe, total free glucose values measured in washed embryo or larval homogenates differed significantly in all PESO-treated groups compared with the hyperglycemic control exposed to 2% D-glucose, regardless of concentration (*p* < 0.05 or *p* < 0.001; Figure 4). Notably, at 48 hpe, corresponding to a glucose-free recovery phase in which all embryos were maintained in glucose-free embryo medium, only embryos previously treated with PESO at 19.1 µg/mL exhibited significantly lower total free glucose values than the 2% D-glucose control group (*p* < 0.05).

Moreover, at 96 hpe, embryos previously treated with PESO displayed lower total free glucose values in washed homogenates than those measured in both the 2% D-glucose group and the embryo-medium control. This response was more evident at the higher PESO concentration (19.1 µg/mL), suggesting a sustained reduction in the measured free glucose signal during the glucose-free recovery interval.

**Figure 5:**
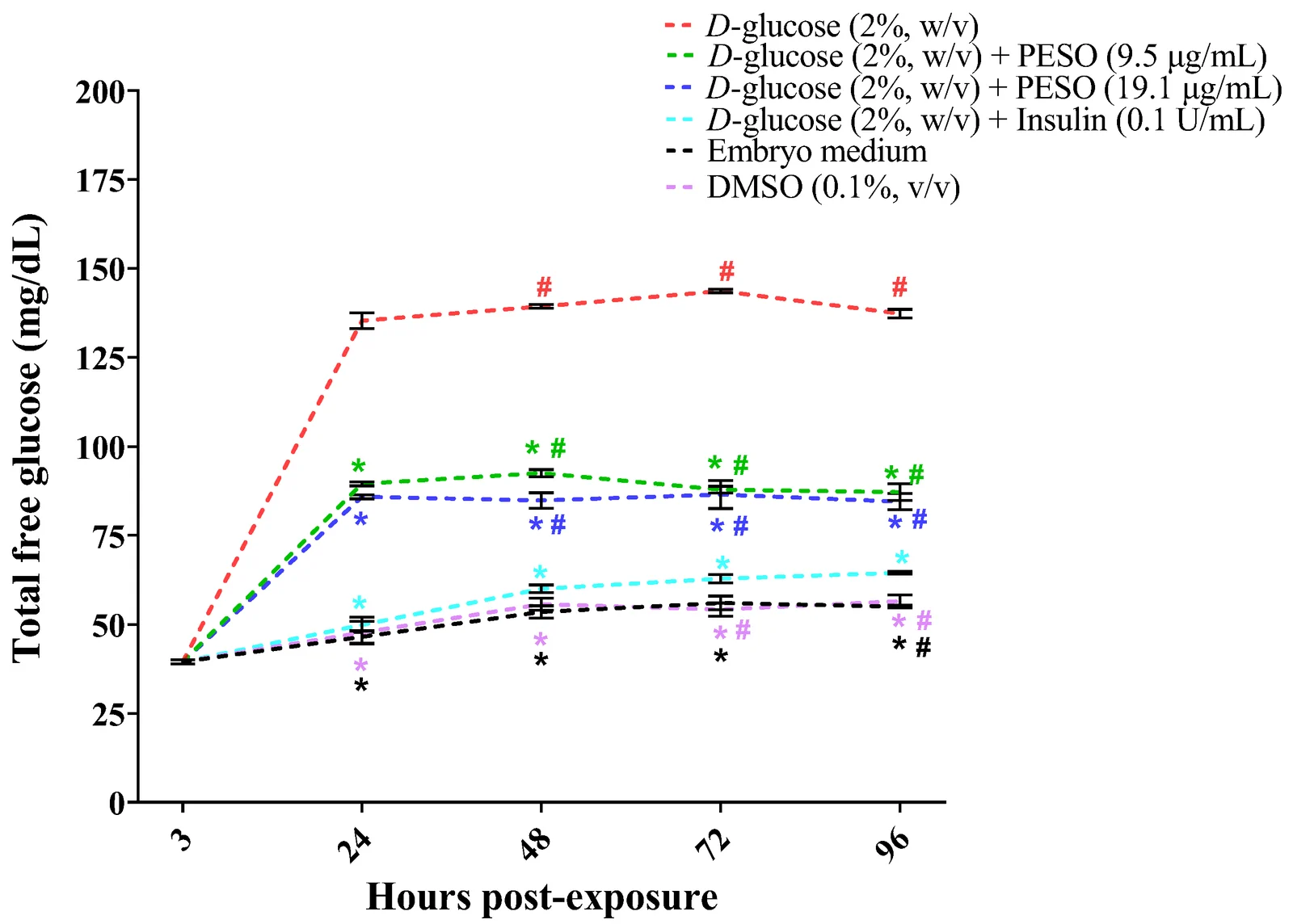
Effect of *Passiflora edulis* seed oil (PESO) on total free glucose measured in washed embryo or larval homogenates under continuous hyperglycemic conditions in *Danio rerio*. Insulin (0.1 U/mL) was included as a pharmacological positive control in the continuous protocol to provide a reference under sustained hyperglycemic exposure. Glucose concentrations were measured at 3, 24, 48, 72, and 96 h post-exposure. PESO was tested at 9.5 and 19.1 µg/mL, and insulin (0.1 U/mL) was included as a positive control. Data were analyzed by two-way ANOVA, followed by Dunnett’s post hoc test vs. *D*-glucose 2% and vs. Insulin 0.1 U/mL group. Significance is indicated as * *p* ≤ 0.05 vs. *D*-glucose and # *p* ≤ 0.05 vs. insulin; symbols are color-coded by treatment group and positioned for clarity. Data are expressed as mean ± SEM (*n* = 3).

Continuous exposure of *Danio rerio* embryos to 2% D-glucose resulted in a significant increase in total free glucose values measured in washed embryo or larval homogenates from 24 hpe onward, with elevated values maintained through 96 hpe. Treatment with PESO at 9.5 and 19.1 µg/mL significantly reduced the measured total free glucose values compared with the 2% D-glucose group at all evaluated time points from 24 hpe (*p* ≤ 0.05). In both PESO-treated groups, the measured values remained below 90 mg/dL throughout the experimental period; however, these values represent glucometer readings obtained from homogenates and should not be interpreted as a systemic normoglycemic range (Figure 5). At 96 hpe, PESO reduced total free glucose values by approximately 36.5% and 38.5% at 9.5 and 19.1 µg/mL, respectively, relative to the D-glucose group, whereas insulin treatment (0.1 U/mL) produced a greater reduction of approximately 53.0%.

### 2.4. Effects of PESO on Oxidative Stress

**Figure 6:**
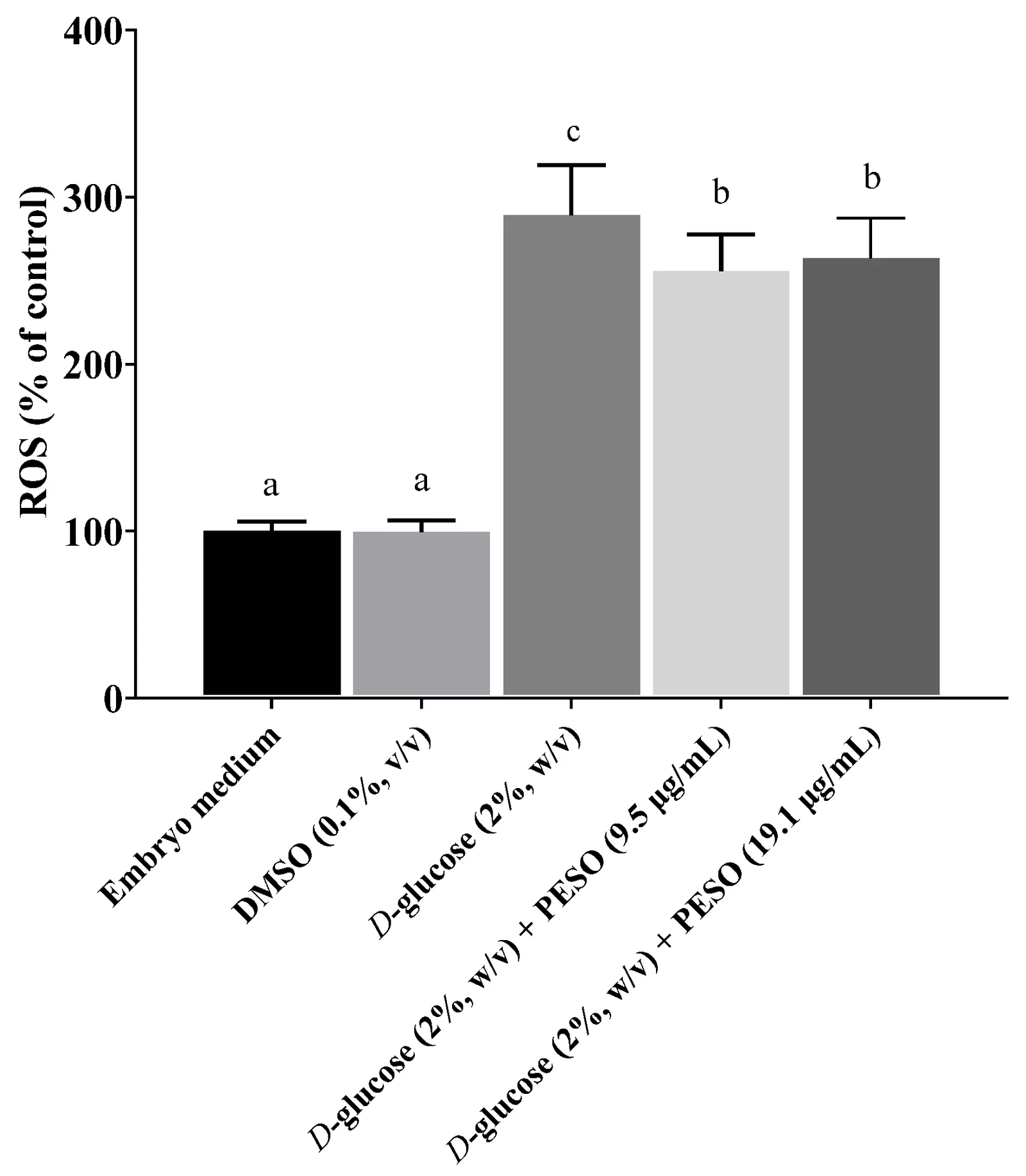
Effect of *P. edulis* seed oil (PESO) on reactive oxygen species (ROS) release in zebrafish larvae exposed to continuous hyperglycemia. ROS levels were quantified after 96 h of exposure to 2% *D*-glucose. Embryo medium was used as the control. Data is presented as mean ± SEM. Statistical analysis was performed using one-way ANOVA followed by Tukey’s post hoc test. Different letters denote statistically significant differences among groups (*p* ≤ 0.05).

Continuous exposure of *Danio rerio* embryos to 2% (*w*/*v*) *D*-glucose for 96 h resulted in significantly higher levels of ROS compared with all other experimental groups, including the negative control (embryo medium), solvent control (0.1% dimethylsulfoxide—DMSO), and PESO-treated groups (9.5 and 19.1 µg/mL) (*p* ≤ 0.05). Treatment with PESO at both concentrations significantly attenuated ROS production relative to the hyperglycemic group (*p* ≤ 0.05). However, ROS levels in PESO-treated larvae remained significantly higher than those observed in the control groups (Figure 6).

### 2.5. Expression Profiles of Selected Antioxidant-Related

**Figure 7:**
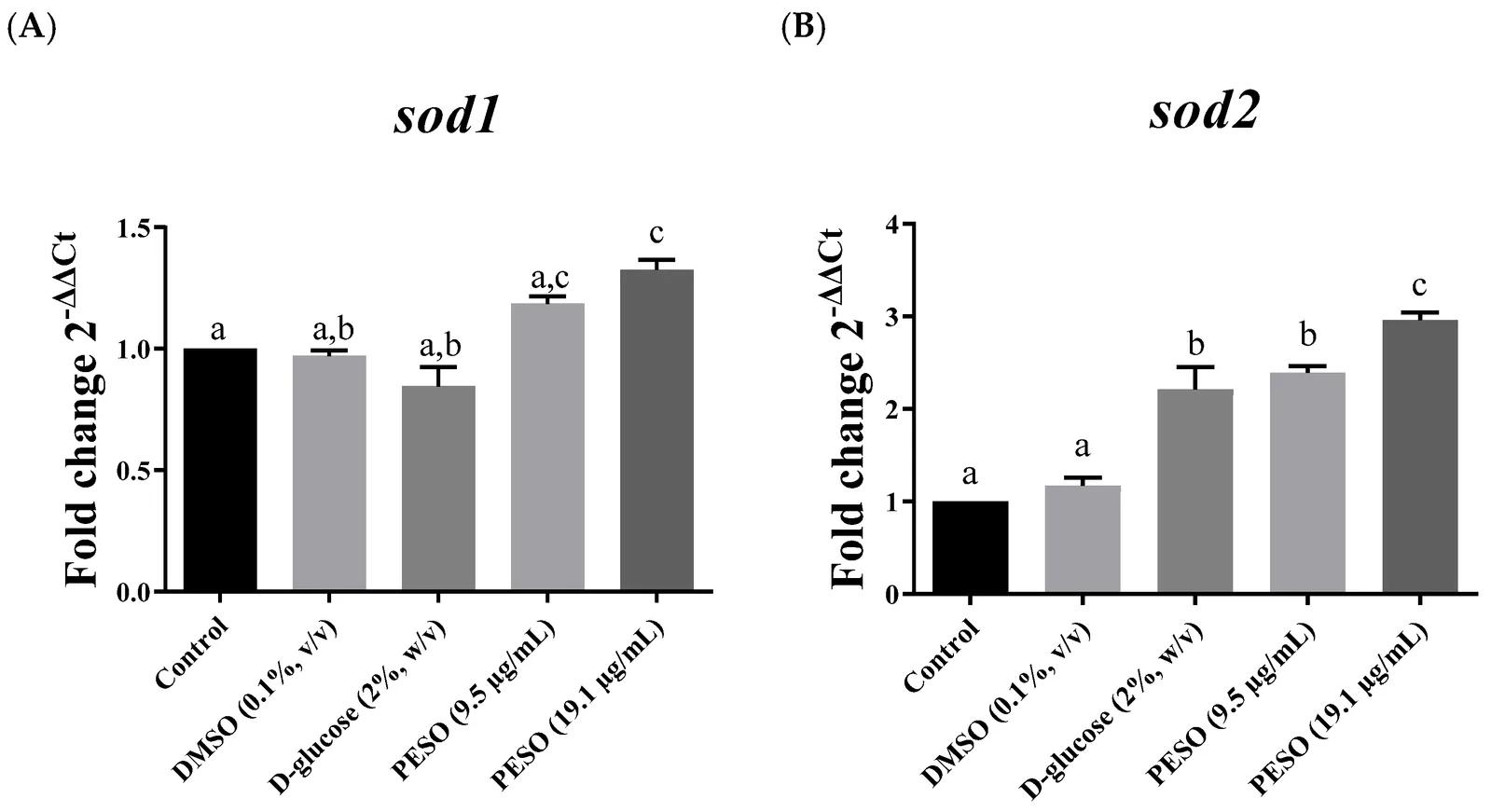

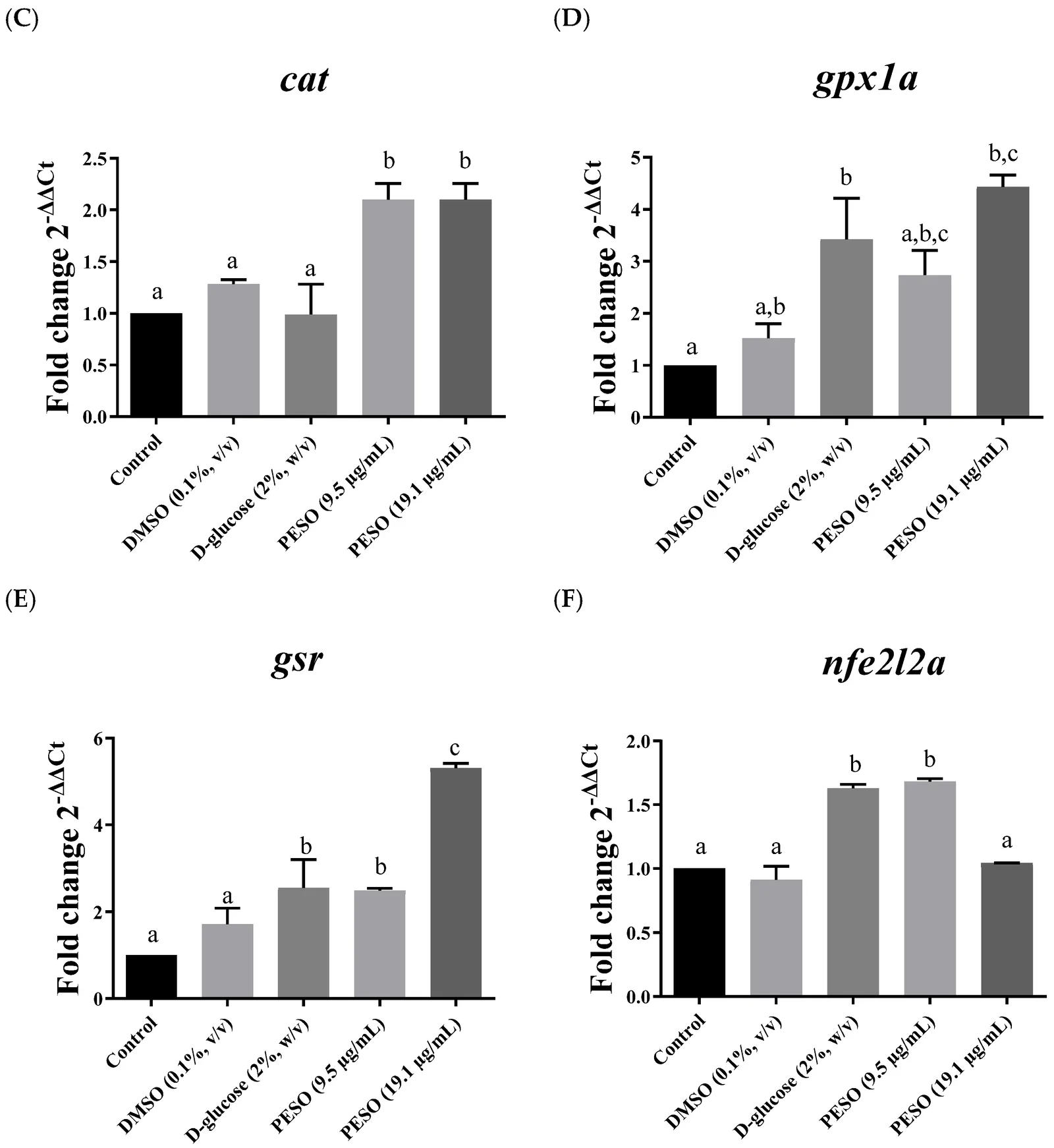
Relative transcript abundance of selected antioxidant-related genes in zebrafish embryos exposed to DMSO (0.1%), 2% D-glucose, or PESO (9.5 and 19.1 µg/mL) for 96 hpe. Genes analyzed: (**A**) *sod1* (superoxide dismutase 1), (**B**) *sod2* (superoxide dismutase 2), (**C**) *cat* (catalase), (**D**) *gpx1a* (glutathione peroxidase 1a), (**E**) *gsr* (glutathione-disulfide reductase), and (**F**) *nfe2l2a* (nuclear factor erythroid 2-related factor 2). Data are presented as mean ± SEM. Statistical analysis was performed using one-way ANOVA followed by Tukey’s post hoc test. Different letters indicate statistically significant differences among groups (*p* ≤ 0.05).

Among the analyzed antioxidant-related genes, distinct expression profiles were observed under hyperglycemic conditions depending on PESO concentration. The genes *superoxide dismutase 2* (*sod2*), *glutathione peroxidase 1a* (*gpx1a*), and *glutathione reductase* (*gsr*) showed significantly higher expression in hyperglycemic embryos treated with PESO at 19.1 µg/mL (*p* ≤ 0.05). In contrast, *catalase* (*cat*) and *superoxide dismutase 1* (*sod1*) were significantly upregulated in hyperglycemic embryos treated with PESO at both 9.5 and 19.1 µg/mL (*p* ≤ 0.05). Regarding the transcriptional regulator *nuclear factor erythroid 2 related factor 2* (*nfe2l2a*), increased expression was observed in the hyperglycemic group (2% *D*-glucose) and in hyperglycemic embryos treated with PESO at 9.5 µg/mL (*p* ≤ 0.05) (Figure 7).

## 3. Discussion

Embryogenesis constitutes a critical period of development during which exposure to adverse metabolic conditions, such as hyperglycemia, can compromise embryonic metabolism and contribute to oxidative stress, inflammation, and long-term developmental consequences [11,16,17]. In humans, hyperglycemia during this period, although managed with insulin therapy, limits the ability to effectively modulate associated inflammatory and redox processes [16,18]. This scenario highlights therapeutic gaps and reinforces the need for strategies that act in an integrated manner across these pathophysiological axes. In this context, the results of this study demonstrate that PESO, despite being a complex lipophilic matrix derived from an agro-industrial by-product, exhibits an adequate safety profile and exerts relevant modulatory effects on glycemia, oxidative stress, and the expression of antioxidant-related genes.

The chemical profile of PESO also supports its biological relevance. In the present study, linoleic acid (C18:2) was the predominant fatty acid, followed by palmitic and stearic acids. Similar findings have been reported for *P. edulis* seed oil, with linoleic acid consistently identified as the major fatty acid [4,19]. The predominance of linoleic acid is relevant, given that polyunsaturated fatty acids may influence membrane organization, redox homeostasis, and metabolic signaling pathways [20,21,22]. However, the chemical characterization performed in the present study was restricted to fatty acid methyl esters. Therefore, the presence and potential contribution of other minor constituents to the observed biological responses cannot be established from the present data.

In toxicological assessments, the determination of the LC_50_ is a fundamental parameter for contextualizing the biological safety of compounds in embryonic models. In this regard, the essential oil of *Zingiber ottensii* Valeton showed an LC_50_ of 1.003 µg/mL in zebrafish embryos [23], which was associated with pronounced developmental abnormalities, including morphological defects, reduced hatching rate, and decreased heart rate. Similarly, the toxicity of different essential oils has been evaluated, with reported LC_50_ values of 3.7 µg/mL for lemongrass, 14.4 µg/mL for thyme, and 5.3 µg/mL for oregano, along with embryonic developmental alterations such as reduced growth, spinal deformities, pericardial edema, decreased eye size, and failure of swim bladder inflation [24]. In contrast, *Pogostemon cablin* oil (patchouli oil) exhibited an LC_50_ of approximately 120 ppm (120 µg/mL) after 72 h of exposure, indicating an intermediate toxicity profile, with a progressive increase in mortality observed only at higher concentrations [25].

Since toxicological evidence involving fixed oils in zebrafish embryos remains limited, comparisons with essential oils were also considered to provide a broader perspective on the embryotoxic potential of plant-derived lipid compounds. Similarly, the LC_50_ reported for *Pogostemon cablin* oil (120 µg/mL at 72 h) was closer to that observed for PESO, which may be partially attributed to similarities in their lipid-rich composition [25]. Comparisons between PESO and essential oils should be interpreted cautiously. Fixed oils are predominantly composed of triglycerides and long-chain fatty acids, whereas essential oils consist mainly of volatile, low-molecular-weight secondary metabolites such as mono- and sesquiterpenes [26]. While both fixed and essential oils may exert antioxidant and metabolic effects, essential oils often exhibit greater biological activity, due to their lipophilicity, which facilitates membrane permeation and interaction with multiple molecular targets [27]. Consequently, LC_50_ values obtained for fixed and essential oils should not be analyzed as directly equivalent or used to establish the relative safety of these oil categories. Essential oil studies were included only to provide a broad toxicological context because embryotoxicity data for fixed plant oils remain limited. Moreover, the developing zebrafish possesses stage-dependent detoxification and antioxidant-defense capacities, which may influence susceptibility to different chemical matrices. Therefore, the LC_50_ value observed for PESO should be evaluated within the specific experimental conditions of the present study rather than as evidence that fixed oils are generally better tolerated than essential oils.

With respect to developmental outcome, several studies have demonstrated that exposure to fixed and/or essential oils during zebrafish embryogenesis is frequently associated with pronounced developmental abnormalities, including reduced hatching rates, decreased heart rate, impaired growth, spinal deformities, pericardial edema, reduced eye size, and failure of swim bladder inflation [23,24,25]. In the present study, embryos exposed to sublethal concentrations of PESO (1/10 and 1/20 of the LC_50_) exhibited only mild developmental alterations, mainly characterized by subtle pigmentation changes, discrete pericardial edema, and occasional incomplete yolk sac absorption. In contrast, embryos subjected to hyperglycemic conditions displayed markedly more severe developmental abnormalities, including pronounced pericardial edema, pigmentation alterations, malformations of the tail and head, impaired yolk sac absorption, and an overall delay in embryonic development.

The predominance of developmental abnormalities under hyperglycemic conditions is consistent with previous reports demonstrating that elevated glucose levels exert direct deleterious effects on zebrafish embryos. In particular, hyperglycemia has been associated with ocular defects, including alterations in retinal layer thickness, increased macrophage infiltration, and reductions in Müller glia and retinal ganglion cells, highlighting the susceptibility of neural and sensory tissues to glucose-induced metabolic stress during early development [28].

*D*-glucose has been widely employed as an exogenous agent to induce hyperglycemic conditions during zebrafish embryogenesis, at concentrations comparable to those reported in animal models and indicative of glycemic fluctuations observed under diabetic conditions [28,29]. The zebrafish model is particularly well-suited for investigating hyperglycemia-related developmental disturbances, as embryos exhibit fluctuating hyperglycemia within clinically relevant ranges and develop externally, allowing direct exposure to glucose [30,31]. This characteristic circumvents the placental barrier present in mammalian models, which can modulate glucose transfer and confound the direct assessment of embryonic effects. While mammalian systems remain essential for understanding complex maternal–fetal interactions, zebrafish embryos offer a unique and powerful platform for early mechanistic investigations of hyperglycemia-induced toxicity and developmental disruption [32,33].

Corroborating the findings discussed above, exposure to hyperglycemic conditions emerged as a major developmental stressor, inducing morphological abnormalities during early embryogenesis and being accompanied by higher total free glucose values in washed embryo or larval homogenates and increased ROS production. Sustained exposure to 2% D-glucose aggravated oxidative stress in zebrafish embryos, reinforcing the deleterious effects of the glucose-enriched microenvironment. Notably, co-exposure to PESO consistently attenuated these alterations under both pulsatile and continuous protocols, reducing the measured total free glucose values and limiting ROS accumulation compared with the hyperglycemic control. Under continuous exposure, the effect of PESO was less pronounced than that of insulin. Collectively, these findings support a modulatory effect of PESO on total free glucose measured in washed homogenates, together with attenuation of oxidative stress, rather than demonstrating a direct systemic hypoglycemic action [28,34,35].

From a mechanistic perspective, the concomitant reductions in total free glucose values and ROS accumulation raise the hypothesis that the lipid composition of PESO may contribute to the observed metabolic and redox-related responses. Linoleic and oleic acids, as well as minor constituents such as tocopherols and phytosterols, had been reported to influence energy- and redox-related processes in other experimental contexts [4]. However, the individual contribution of these constituents was not evaluated in the present study. Moreover, mitochondrial function, cellular energy metabolism, insulin signaling, glucose transport, and gluconeogenic pathways were not assessed. Therefore, the present findings do not demonstrate that PESO directly modulates these pathways or definitively improves the intracellular redox environment. Rather, they indicate an association between PESO exposure and lower total free glucose values, partial attenuation of ROS accumulation, and changes in selected antioxidant-related transcripts under glucose-induced metabolic stress. Further mechanistic studies are required to determine the cellular pathways underlying these responses [34,36].

Because zebrafish embryos were directly exposed to glucose and PESO in the absence of placental mediation, the observed responses occurred without a maternal or placental interface. Nevertheless, this experimental design does not establish a direct molecular protective mechanism of PESO. In addition, ROS levels were assessed only at 96 hpe; therefore, the present findings describe the oxidative status at the end of the continuous exposure period and do not establish the temporal onset or progression of ROS modulation. Time-resolved analyses, together with direct assessments of mitochondrial and metabolic function, will be required to characterize the mechanisms and dynamics of this response.

Within the zebrafish model, which enables direct embryonic exposure to glucose in the absence of placental modulation, PESO treatment was associated with lower total free glucose values and partial attenuation of ROS accumulation under glucose-induced metabolic stress. The concomitant occurrence of these responses suggests a possible relationship between free glucose reduction and redox modulation. However, because mitochondrial function, cellular energy metabolism, insulin signaling, glucose transport, and gluconeogenic pathways were not evaluated, the present findings do not establish a direct protective mechanism or an integrated mode of action. It is important to note that ROS levels were assessed only at 96 hpe; therefore, the present findings reflect the oxidative status at the end of the continuous exposure period and do not establish the temporal onset or progression of ROS modulation by PESO. Time-resolved analyses at earlier developmental stages will be required to characterize the dynamics of this response. These findings provide a basis for the subsequent discussion of the changes observed in selected antioxidant-related transcripts under glucose-induced metabolic stress [28].

Consistent with the previously observed attenuation of ROS levels, PESO treatment was associated with distinct concentration-related expression profiles among the selected antioxidant-related genes, suggesting the differential involvement of components of endogenous antioxidant defense. The upregulation of *sod1* and *cat* (Figure 7) at both tested concentrations suggests the involvement of cytosolic antioxidant defenses across the evaluated PESO exposure levels. The corresponding enzymes constitute important components of the first line of defense against oxidative stress, with SOD1 catalyzing the dismutation of superoxide radicals and CAT preventing hydrogen peroxide accumulation following superoxide dismutation. Their responsiveness at the lower PESO concentration suggests that these antioxidant defenses may be recruited under glucose-induced oxidative stress even at the lower exposure level [37,38].

In contrast, the selective upregulation of *sod2*, *gpx1a*, and *gsr* at 19.1 µg/mL suggests the additional involvement of mitochondrial and glutathione-dependent antioxidant mechanisms at the higher PESO concentration. SOD2 operates within the mitochondrial compartment, detoxifying superoxide generated by the electron transport chain, whereas GPx1a and GSR are central components of the glutathione system, contributing to peroxide reduction and maintenance of intracellular redox homeostasis through glutathione recycling. The preferential upregulation of these genes at 19.1 µg/mL therefore indicates a concentration-associated expression profile involving mitochondrial and glutathione-dependent antioxidant defenses under glucose-induced oxidative stress. Notably, *nfe2l2a* was upregulated at 9.5 µg/mL, which is consistent with the role of Nrf2 as a redox-sensitive transcriptional regulator involved in antioxidant responses. Together, these findings indicate that, under glucose-induced metabolic stress, PESO treatment was associated with coordinated but distinct concentration-related expression profiles among the selected antioxidant-related genes. The lower concentration was associated primarily with *nfe2l2a*, *sod1*, and *cat* responses, whereas the higher concentration was additionally associated with *sod2*, *gpx1a*, and *gsr* expression [39,40,41].

Although PESO exhibited promising biological effects, certain methodological constraints must be considered. This investigation was conducted solely using a zebrafish embryonic model, which, despite being highly informative for early developmental, metabolic, and redox-related processes, does not encompass the full physiological complexity of mammalian systems. Notably, the absence of placental mediation in zebrafish embryos limits the direct translation of these findings to mammalian gestational contexts, in which the placenta plays a pivotal role in regulating glucose transport, redox homeostasis, and fetal metabolic programming. Accordingly, the translational implications of the protective responses observed under hyperglycemic conditions should be interpreted with appropriate caution. Furthermore, the experimental approach was restricted to exposure during early embryogenesis and did not address long-term, chronic, or reproductive outcomes.

The pulsatile and continuous protocols were designed to address distinct experimental questions. The pulsatile protocol evaluated embryonic responses during alternating periods of glucose exposure and glucose-free recovery without pharmacological intervention, whereas insulin was included in the continuous protocol as a pharmacological reference under sustained hyperglycemic exposure. Consequently, the absence of an insulin-treated group in the pulsatile protocol precludes the establishment of a common pharmacological benchmark across the two paradigms, and the magnitude of PESO-associated responses should not be directly compared between them.

Regarding the molecular analyses, although *actb* was selected after comparison with *elfa* and *gapdh*, normalization using multiple validated reference genes could provide additional robustness in future studies. PESO-only groups were included in the embryotoxicity and survival assessments but were not included in the ROS and gene expression experiments. Therefore, the present study cannot determine whether PESO alters basal redox status or antioxidant-related gene expression in embryos maintained without glucose exposure. The redox and transcriptional findings are thus restricted to the effects associated with PESO treatment in the presence of glucose-induced metabolic stress. In addition, the qPCR findings reflect relative transcript abundance and should not be interpreted as direct evidence of increased antioxidant enzyme function. Protein abundance and the enzymatic activities of SOD, CAT, GPx, and GSR were not evaluated, and the GSH/GSSG ratio was not measured. Consequently, the functional consequences of the observed transcriptional changes remain open. Protein abundance and the enzymatic activities of SOD, CAT, GPx, and GSR were not evaluated, and the GSH/GSSG ratio was not measured. Consequently, the functional consequences of the observed transcriptional changes remain to be established. Inflammation is retained only in the general background as a recognized consequence of hyperglycemia and is not attributed as an experimentally demonstrated effect of PESO.

Glucose was quantified as total free glucose in washed embryos or larval homogenates using a portable glucometer. The experimental design included an embryo-medium negative control, a 2% D-glucose hyperglycemic control, and an insulin-treated positive control, and embryos or larvae were rinsed three consecutive times before homogenization. Nonetheless, the efficacy of exogenous-glucose elimination was not autonomously substantiated by a washing-gradient experiment, and the glucometer readings were not corroborated by an independent enzyme-based quantification technique. Consequently, the findings are to be regarded as comparative assessments of total free glucose in washed homogenates instead of a direct or specific quantification of blood glucose or intracellular glucose. Future studies incorporating these methodological refinements, together with long-term evaluations in complementary experimental models, will be important for a more comprehensive assessment of both safety and efficacy, particularly when considering the potential application of bioactive compounds in gestational or pediatric contexts.

## 4. Materials and Methods

### 4.1. Chemical

All reagents were of analytical grade. DMSO, 3,4-dichloroaniline, *D*-glucose, phosphate-buffered saline (PBS), 2′,7′-dichlorodihydrofluorescein diacetate (H_2_DCFDA), and nuclease-free water were obtained from Sigma-Aldrich (St. Louis, MO, USA). RNAlater^®^ Solution and PureLink™ RNA Mini Kit were purchased from Invitrogen/Thermo Fisher Scientific (Waltham, MA, USA). The High-Capacity cDNA Reverse Transcription Kit and SYBR^®^ Green Master Mix were obtained from Applied Biosystems (Foster City, CA, USA). The QuantiNova^®^ SYBR Green PCR Kit was acquired from Qiagen (Hilden, Germany).

Insulin (human recombinant) was obtained from Novo Nordisk (Bagsværd, Denmark). Accu-Chek Active^®^ glucometer and test strips were supplied by Roche Diagnostics (Mannheim, Germany). Commercial zebrafish feed (Alcon Basic^®^, Camboriú, Brazil) and *Artemia salina* cysts (Artemia do RN^®^, Natal, Brazil) were also used. Gene-specific primers for *sod1*, *sod2*, *cat*, *gpx1a*, *gsr*, *nfe2l2a*, and actb were synthesized by Integrated DNA Technologies (IDT, Coralville, IA, USA).

### 4.2. Preparation of Passiflora edulis Seed Oil (PESO)

Seeds of *Passiflora edulis* were obtained from organically cultivated sour passion fruits supplied by Paraíso Agrícola Farm, a member of the local family farming cooperative in Matriz do Camaragibe, Alagoas, Brazil (9°09′24″ S, 35°31′45″ W). The botanical material was identified by researchers from the Herbarium MAC of the Instituto do Meio Ambiente do Estado de Alagoas, Brazil, and a voucher specimen was deposited under registration number MAC 78263, corresponding to *Passiflora edulis* Sims (Passifloraceae). The use of Brazilian genetic heritage was registered in SisGen under code A1CA908.

After harvest, the seeds were manually separated from the pulp, thoroughly washed under running water, and dried in a forced-air oven at 50 °C for 48 h. The dried seeds were then mechanically pressed using a 15-t hydraulic press (Marcon, MPH-15, Marília, Brazil) operated at ambient temperature (20–25 °C) without external heating or cooling. The resulting crude PESO was left undisturbed for ~48 h to allow sedimentation. The clarified upper fraction was carefully decanted, avoiding contact with the sediment layer, transferred to sterile amber containers, and stored at 4 °C until use.

#### 4.2.1. Fatty Acid Transesterification

FAMEs were prepared according to the method described by Joseph and Ackman [42]. Briefly, PESO (25.0 ± 0.1 mg) was mixed with 1.5 mL of 0.5 mol/L NaOH in methanol and heated at 100 °C under stirring for 5 min. After cooling, 2.0 mL of 12% (*v*/*v*) BF_3_ in methanol was added, and the reaction mixture was maintained at 100 °C under stirring for 30 min. Subsequently, 1 mL of hexane was added, followed by vigorous mixing for 30 s and the addition of 5 mL of saturated NaCl solution. The mixture was refrigerated until complete phase separation. The upper organic phase was collected, combined with an additional 1 mL of hexane, concentrated, and stored at −18 °C under an N_2_ atmosphere until chromatographic analysis.

#### 4.2.2. Gas Chromatography with Flame Ionization Detection (GC-FID)

FAMEs were analyzed using a GC-2010 Plus gas chromatograph equipped with a flame ionization detector (Shimadzu Corporation, Kyoto, Japan) and an NTS capillary column (30 m × 0.25 mm i.d. × 0.25 μm). Nitrogen was used as a carrier gas at a column flow rate of 0.71 mL/min. The oven temperature was initially set at 120 °C and increased to 300 °C at 6 °C/min. Analyses were performed in split mode by injecting 1 μL of the esterified oil solution prepared in HPLC-grade hexane.

#### 4.2.3. Gas Chromatography–Mass Spectrometry (GC-MS)

GC-MS analysis was performed using a Shimadzu QP-2010 gas chromatograph coupled to a mass spectrometer and fitted with an SH-5MS capillary column (30 m × 0.25 mm i.d. × 0.25 μm; Shimadzu). Helium was used as the carrier gas at a flow rate of 0.71 mL/min. Mass spectra were acquired in electron ionization mode at 70 eV. The oven temperature was initially maintained at 125 °C for 5 min, increased to 300 °C at 6 °C/min, and held at this temperature for 10 min. A 1 μL aliquot of the esterified oil solution prepared in HPLC-grade hexane was injected in splitless mode.

#### 4.2.4. Identification and Quantification of Fatty Acid Methyl Esters

FAMEs were identified by comparing their retention times with authentic fatty acid methyl ester standards and by analyzing their mass fragmentation patterns and retention indices, which were compared with spectral library data, including NIST and Wiley databases. Quantification was performed from GC-FID peak areas using methyl palmitate as an external reference standard. The calibration curve was constructed using methyl palmitate solutions ranging from 10 to 75 ppm, and FAME concentrations were expressed as µg/mL. Relative abundance was calculated as the percentage of the total chromatographic peak area.

### 4.3. Animal Model

All procedures involving adult zebrafish and their embryos were approved by the Animal Use Ethics Committee of the Federal University of Alagoas (UFAL; protocol no. 13/2022) and were conducted in accordance with applicable guidelines for the care and use of laboratory animals. Acute toxicity was assessed using the Fish Embryo Acute Toxicity (FET) test in accordance with OECD Guideline 236 [43]. The median lethal concentration (LC_50_) was determined and used to select sublethal concentrations for the subsequent embryotoxicity, metabolic, biochemical, and molecular assays [44].

Adult zebrafish (*Danio rerio*, AB strain) were maintained in a recirculating aquaculture system under controlled conditions (28 °C; pH 6.7–7.2; dissolved oxygen approximately 6 mg/L; conductivity 1600 µS/cm; total ammonia < 0.01 mg/L). Fish were kept at a density of 2 fish/L under a 14 h light/10 h dark photoperiod (lights on at 07:00; 190 lux) and were fed three times daily with *Artemia salina* nauplii and a commercial diet containing 60% protein and 15% lipids.

**Figure 8:**
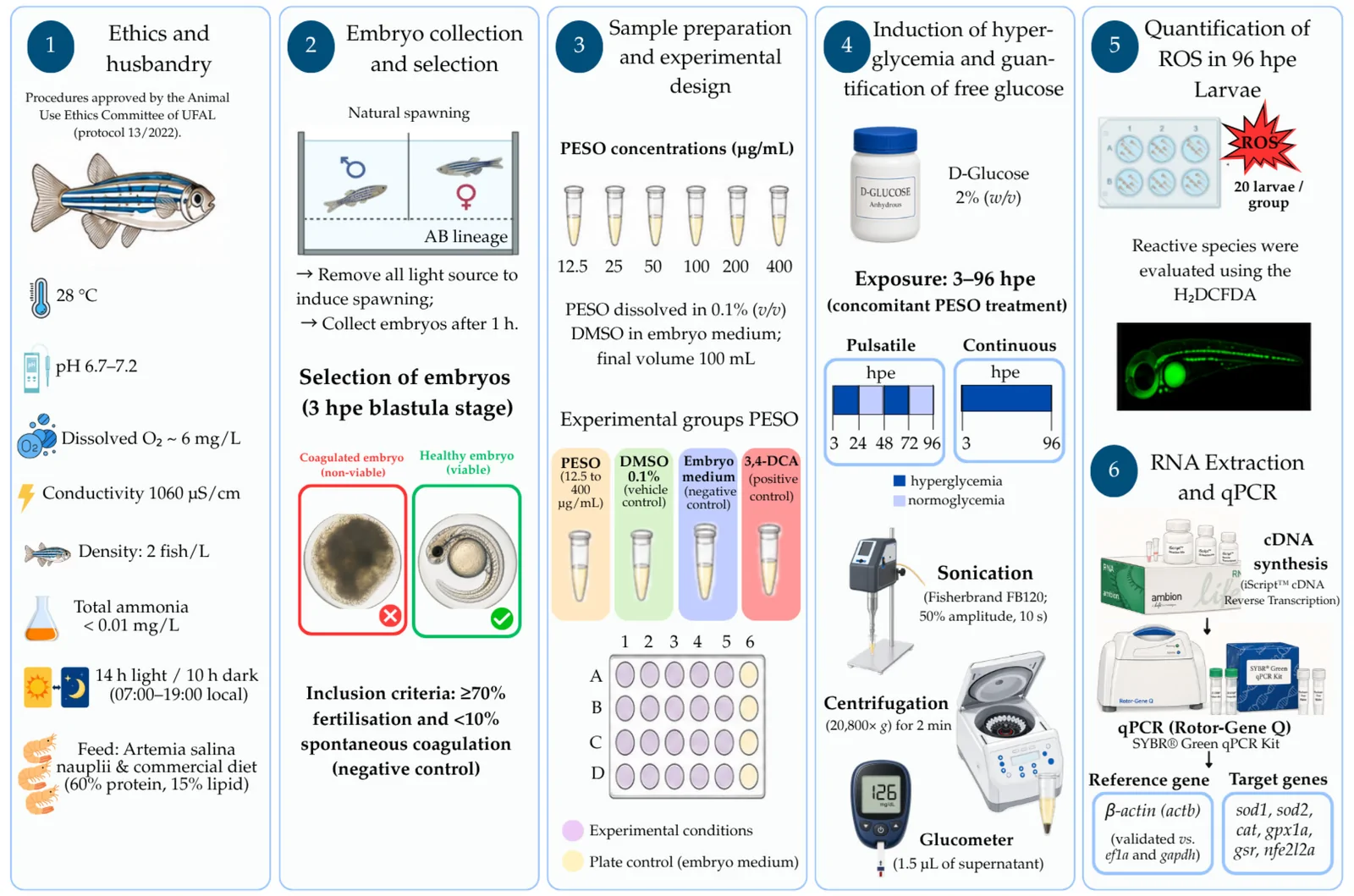
Schematic workflow of the experimental design used to evaluate the effects of PESO in *Danio rerio*.

Embryos were obtained by natural spawning. Adult breeders were placed in mating tanks at a male-to-female ratio of 3:2 and separated overnight by a perforated translucent divider that permitted visual and chemical contact. At light onset, the divider was removed, and embryos were collected after 1 h. Fertilization and developmental stage were confirmed under a stereomicroscope (40×). Only normally developing embryos at the blastula stage (2–3 h post-exposure, hpe) from batches showing ≥70% viability and <10% spontaneous coagulation in the negative control were included. The experimental workflow is summarized in Figure 8.

The figure summarizes the main experimental steps, including embryo collection and selection, exposure to PESO and control groups, acute toxicity assessment, hyperglycemia induction, and subsequent biochemical and molecular analyses.

#### 4.3.1. Sample Preparation, Experimental Design, and Sample-Size Determination

PESO working solutions were prepared in embryo medium using DMSO as the solvent, with a final DMSO concentration of 0.1% (*v*/*v*). All treatment and control solutions were freshly prepared. The controls were selected according to the objective of each assay: embryo medium served as the negative control, 0.1% DMSO as the solvent control, 2% (*w*/*v*) D-glucose as the hyperglycemic control, 3,4-dichloroaniline (4 mg/L) as the positive control for embryotoxicity, and insulin (0.1 U/mL) as the glycemic recovery control.

Three independent biological experiments were performed. In the acute toxicity and embryotoxicity assays, 20 embryos were allocated to each experimental group in each independent experiment, resulting in 60 embryos per group. Embryos were maintained individually in 24-well plates (one embryo per well), and four additional wells containing embryos in embryo medium were included as internal plate controls. For these assays, the individual embryo was considered the experimental unit. For glucose, ROS, and gene expression analyses, embryos or larvae were pooled, and each independently prepared pool was considered one experimental unit. Technical replicate readings were averaged and were not treated as independent biological observations.

Sample sizes were determined a priori in the experimental protocol approved by the institutional ethics committee. The number of embryos used in the toxicity and embryotoxicity assays was based on OECD Guideline 236 [40], whereas the pool sizes used for biochemical and molecular analyses were based on previously published zebrafish studies and on the minimum amount of biological material required for reliable glucose, ROS, and RNA measurements. The design also followed the reduction principle of the 3Rs. No post hoc adjustment of sample size was performed [43].

#### 4.3.2. Acute Toxicity Assessment and Determination of LC_50_ in *Danio rerio* Embryos

Acute toxicity was evaluated in embryos exposed to PESO at 12.5, 25, 50, 100, 200, or 400 µg/mL for 96 h. At 2–3 hpe, viable embryos were randomly selected from the pooled spawn, rinsed, and transferred individually to 24-well plates containing 2 mL of the corresponding test solution per well. The assay included embryo medium as the negative control, 0.1% (*v*/*v*) DMSO as the solvent control, and 3,4-dichloroaniline (4 mg/L) as the positive control. Each group contained 20 embryos per independent experiment.

A semi-static exposure system was used, with complete renewal of all solutions every 24 h. Embryos were examined daily under a stereomicroscope throughout the 96-h exposure. Lethality was defined according to OECD Guideline 236 by the presence of at least one of the following endpoints: embryo coagulation, absence of somite formation, failure of tail detachment from the yolk sac, or absence of cardiac activity [43].

Hatching and non-lethal developmental alterations were also recorded but were not included in the LC_50_ calculation.

Cumulative mortality at 96 h was used to construct concentration–response curves. The LC_50_ was estimated by nonlinear regression, and concentrations corresponding to 1/10 and 1/20 of the LC_50_ were selected for the subsequent embryotoxicity and functional assays.

#### 4.3.3. Embryotoxicity and Survival Assessment up to 144 Hpe

The developmental safety of the two selected sublethal PESO concentrations (9.5 and 19.1 µg/mL, corresponding to 1/20 and 1/10 of the LC_50_, respectively) was evaluated in an independent 144 hpe embryotoxicity assay. The experimental groups were embryo medium (negative control), 0.1% (*v*/*v*) DMSO (solvent control), 2% (*w*/*v*) D-glucose (hyperglycemic control), 3,4-dichloroaniline (4 mg/L; positive embryotoxicity control), PESO 9.5 µg/mL, and PESO 19.1 µg/mL. Each group comprised 20 embryos per independent experiment, and three independent biological experiments were conducted.

Exposure began at 2–3 hpe and was conducted under semi-static conditions, with complete renewal of the corresponding solutions every 24 h. Survival was recorded at the start of exposure and at 3, 24, 48, 72, 96, 120 and 144 hpe. The same OECD lethality criteria defined for the acute toxicity assay were applied, and non-viable embryos or larvae were removed when detected.

Developmental endpoints were assessed under a stereomicroscope and included somite formation, eye development, spontaneous movement, pigmentation, pericardial oedema, cranial and caudal malformations, yolk-sac malformation or incomplete absorption, and general developmental delay. The occurrence of each abnormality was summarized across the three independent experiments using the 0–5 ordinal scale employed for heatmap visualization, in which 0 indicated absence, and 5 represented the highest observed frequency.

#### 4.3.4. Induction of Hyperglycemia and Quantification of Total Free Glucose in Washed *Danio rerio* Embryo and Larval Homogenates

Hyperglycemia was induced by exposing embryos to 2% (*w*/*v*) D-glucose from 3 to 96 hpe. The experimental groups included embryo medium, 0.1% (*v*/*v*) DMSO, 2% D-glucose, 2% D-glucose plus PESO at 9.5 or 19.1 µg/mL, and, in the continuous-exposure experiment, 2% D-glucose plus insulin (0.1 U/mL; 2 h exposure) as an insulin-treated positive control. Embryos were maintained in 6-well plates containing 10 mL of the corresponding solution per well [28]. For each treatment and sampling time, each biological replicate consisted of a pool of 60 embryos or larvae.

Two exposure paradigms, designed to address distinct experimental questions, were evaluated. The pulsatile protocol was conceived as an exploratory exposure–recovery paradigm to characterize the metabolic responses of zebrafish embryos during alternating periods of glucose exposure and recovery in glucose-free embryo medium, without pharmacological intervention. Embryos were exposed to glucose-containing solutions from 3 to 24 hpe and from 48 to 72 hpe and were maintained in glucose-free embryo medium during the recovery intervals from 24 to 48 hpe and from 72 to 96 hpe. PESO was administered during the corresponding glucose-exposure periods. Because this protocol was intended to evaluate embryo responses during glucose exposure and subsequent recovery without pharmacological interference, insulin was not included. In contrast, the continuous protocol was designed to evaluate PESO under sustained hyperglycemic conditions. Embryos remained exposed to 2% *D*-glucose, alone or in combination with PESO, from 3 to 96 hpe, with complete renewal of all solutions every 24 h. In this protocol, insulin was included as a pharmacological positive control to provide a reference response under continuous glucose exposure.

Independent pools were collected at 3, 24, 48, 72, and 96 hpe. At each sampling time, the exposure medium was completely removed, and embryos or larvae were rinsed three consecutive times with Milli-Q water. Only the washed biological material was transferred to sterile 2 mL tubes, and no exposure medium was included in the homogenates. Embryos or larvae were then euthanized by cooling and lysed by sonication in 200 μL PBS (Fisherbrand FB120; 50% amplitude, 10 s). Homogenates were centrifuged at 14,000 rpm (20,800× *g*) for 2 min, and the supernatants were retained for glucose quantification.

Total free glucose in the supernatants of washed embryo or larval homogenates was measured using an Accu-Chek Active^®^ glucometer (Roche Diagnostics, Rotkreuz, Switzerland). For each biological sample, 1.5 μL of supernatant was applied to the test strip, and three consecutive technical readings were obtained. The mean of the three readings was used as the value for that biological replicate. Three independent biological replicates were analyzed for each treatment and sampling time. The analytical readout was interpreted as total free glucose in the washed homogenate and not as a direct measurement of blood glucose or selective intracellular glucose.

#### 4.3.5. Quantification of ROS in *Danio rerio* Larvae

ROS production was quantified at 96 hpe as a terminal endpoint of the continuous hyperglycemia protocol. The groups comprised embryo medium, 0.1% DMSO, 2% D-glucose, and 2% D-glucose plus PESO at 9.5 or 19.1 µg/mL. For each condition, three independent biological replicates were analyzed, each consisting of a pool of 20 larvae. Larvae were euthanized by cooling, washed three times with ice-cold PBS (pH 7.4), lysed by sonication in 200 μL PBS (Fisherbrand FB120; 50% amplitude, 10 s), and centrifuged at 10,000× *g* for 10 min at 4 °C [45].

Reactive species were detected using 2′,7′-dichlorodihydrofluorescein diacetate (H_2_DCFDA; Thermo Fisher Scientific). For each reaction, 40 μL of supernatant was incubated with 40 μM H_2_DCFDA for 30 min at 37 °C in the dark. Fluorescence was measured using a GloMax^®^ Multi Detection System (Promega Corporation, Madison, WI, USA) at 490 nm excitation and 530 nm emission.

Each biological sample was analyzed in technical quintuplicate, and the mean of the five readings was used as one biological value. ROS levels were expressed as a percentage of the negative control.

#### 4.3.6. RNA Extraction and Quantitative Real-Time Polymerase Chain Reaction (qPCR)

Gene expression analyses were performed at 96 hpe using the same continuous-exposure groups evaluated in the ROS assay. Three independent biological replicates were analyzed per group, each consisting of a pool of 20 larvae. Larvae were euthanized by cooling and immediately preserved in RNAlater^®^ (Thermo Fisher Scientific). Total RNA was extracted using the PureLink™ RNA Mini Kit (Life Technologies, Carlsbad, CA, USA) according to the manufacturer’s instructions, and RNA concentration and purity were assessed using a NanoDrop™ One spectrophotometer (Thermo Fisher Scientific, Waltham, MA, USA).

RNA samples were normalized to 100 ng/µL before cDNA synthesis using the High-Capacity cDNA Reverse Transcription Kit (Applied Biosystems). qPCR reactions were prepared in a final volume of 10 µL containing 10 ng cDNA, 5 µL SYBR^®^ Green Master Mix, 1 µL forward primer, 1 µL reverse primer, 2 µL nuclease-free water, and 1 µL cDNA. Amplification was performed on a Rotor-Gene Q system (Qiagen, Hilden, Germany).

The relative transcript abundance of *sod1*, *sod2*, *cat*, *gpx1a*, *gsr*, and *nfe2l2a* was calculated using the 2^−ΔΔCt^ method [46]. β-Actin (*actb*) [47] was used as the reference gene after empirical comparison with elongation factor 1-alpha (*elfa*) and glyceraldehyde-3-phosphate dehydrogenase (*gapdh*). Among the three candidate genes, *actb* showed the lowest intra-group and inter-group variability under glucose and PESO exposure and was therefore selected for normalization.

The transcriptional panel was selected a priori as a targeted exploratory set representing superoxide dismutation (*sod1* and *sod2*), hydrogen peroxide removal (*cat* and *gpx1a*), glutathione recycling (*gsr*), and redox-responsive transcriptional regulation (*nfe2l2a*). The panel was not intended to provide a comprehensive characterization of the Nrf2–Keap1 regulatory pathway.

All qPCR reactions were performed in technical triplicate, and the mean cycle-threshold value was used for each biological replicate. Primer sequences, accession numbers, expected amplicon sizes, and melting temperatures are provided in Table 2. The reporting of the qPCR procedures followed MIQE recommendations [48,49].

### 4.4. Statistical Analysis

Statistical analyses were performed using GraphPad Prism 9.0 (GraphPad Software, San Diego, CA, USA). Data are presented as mean ± standard error of the mean (SEM), and *p* ≤ 0.05 was considered statistically significant. The value of n represents independent biological experiments or independently prepared biological pools. Technical replicate measurements were averaged before statistical analysis.

For the acute toxicity assay, the LC_50_ and its concentration–response curve were obtained by nonlinear regression using the log(inhibitor) versus normalized response model with a variable slope. Developmental abnormalities in the embryotoxicity assay were described in a heatmap using a semiquantitative ordinal scale ranging from 0 (no observable alteration) to 5 (highest observed severity). Because these scores represented descriptive summaries rather than embryo-level incidence data, no endpoint-specific inferential statistical analysis was performed.

Total free glucose values obtained from washed embryo or larval homogenates in the pulsatile and continuous hyperglycemia experiments were analyzed by two-way ANOVA to assess the effects of treatment, developmental time, and their interaction. Dunnett’s multiple-comparison test was used to compare treatment groups with hyperglycemic control. In the continuous model, a separate Dunnett comparison was also performed against the insulin-treated group.

ROS production and relative gene expression data were analyzed by one-way ANOVA followed by Tukey’s multiple-comparison test to identify differences among experimental groups.

### 4.5. Artificial Intelligence Statement

Generative artificial intelligence was used to assist in creating some graphical elements incorporated into the graphical abstract and the experimental workflow figure (Figure 8), based on detailed author-provided instructions. Figure 8 was subsequently redesigned, assembled, and reviewed by the authors to ensure scientific accuracy and consistency with the experimental design. No AI tools were used in the study design, data collection, analysis, interpretation, or generation of results. AI was also used for minor language refinement.

## 5. Conclusions

The zebrafish embryo model provided an efficient, low-cost, and ethically advantageous platform for the preliminary screening of developmental toxicity and glucose- and redox-related responses to plant-derived oils. Within this experimental framework, PESO displayed a favorable developmental safety profile and reduced total free glucose values in washed embryos or larval homogenates under experimentally induced hyperglycemic conditions during embryogenesis. Under glucose-induced metabolic stress, PESO also partially attenuated ROS accumulation and was associated with the modulation of selected antioxidant-related transcripts. However, because the embryos were directly exposed to glucose in the absence of a maternal organism, placental interface, and maternal metabolic regulation, this model does not reproduce mammalian gestational physiology or maternal–fetal glucose regulation. The findings should therefore be interpreted as preliminary evidence obtained specifically in an experimental model of hyperglycemia during embryogenesis.

Beyond the observed biological outcomes, the present findings also highlight the potential of agro-industrial residues as valuable sources of functional bioactive compounds, in line with sustainability, circular economy, and bioeconomy principles, with prospective benefits for food innovation and public health. To further substantiate these results, future investigations should include in vivo mammalian models to clarify the influence of placental regulation on the metabolic and redox actions of PESO and to assess long-term safety and developmental effects. Additionally, the development of formulation strategies to enhance oil solubility, bioavailability, and targeted delivery will be essential to support translational and nutraceutical applications.

From a nutritional and public health standpoint, the present findings extend beyond experimental observations of redox modulation. Diabetes mellitus remains a growing global concern, disproportionately affecting low- and middle-income settings, where access to comprehensive metabolic follow-up and adjunct therapeutic strategies may be limited. Although pharmacological management remains essential, dietary intervention represents the first-line approach and plays a central role in metabolic regulation. Thus, the identification of plant-derived oils associated with reduced total free glucose accumulation and oxidative imbalance under the tested experimental conditions, particularly those obtained from low-cost agro-industrial residues such as *Passiflora edulis* seeds, supports further investigation of these bioactive lipid matrices as potential ingredients for functional foods or nutritionally targeted formulations.

## Figures and Tables

**Table 1:** Fatty acid composition of *Passiflora edulis* seed oil determined as fatty acid methyl esters.

Peak	RT Min	Compound	Corresponding Fatty Acid	Area %	Concentration µg/mL
1	15.932	Methyl palmitate	Palmitic acid C16:0	11.24	47.35
2	18.754	Methyl linoleate	Linoleic acid C18:2	66.93	282.00
3	18.861	Methyl cis-9-octadecenoate	Oleic acid C18:1 cis-9	17.03	71.74
5	19.271	Methyl octadecanoate	Stearic acid C18:0	4.80	20.21

Fatty acids are reported according to their corresponding methyl ester derivatives. RT: retention time; Area (%): relative chromatographic peak area. Concentrations are expressed as estimated values in µg/mL. Quantification was performed by comparison with the peak area of the methyl palmitate external standard curve (10–75 ppm) using GC-FID.

**Table 2:** Primer sequences for gene expression analysis and associated parameters.

*Gene*	Database Accession Number	Primer Forward (5′–3′)	Primer Reverse (5′–3′)	Size (bp)	Melting Temperature (°C)
*sod1 * ^1^	NM_131294.1	GTAATGTGACCGCTGATGCC	GAATCACCATGGTCCTCCCA	105	60
*sod2 * ^1^	NM_199976.1	TTGGAGGCCATAAAGCGTGA	CCTTTTCAAAGCCCAGCCAG	112	60
*cat * ^1^	NM_130912.2	TCTCCTGATGTGGCCCGATA	GGTTTTGCACCATGCGTTTC	169	60
*gpx1a * ^1^	NM_001007281.2	TAAAACCTGCGTGTTGCCCT	ATAGTTTCGCGGACAGGTCG	97	60
*gsr * ^1^	NM_001020554.1	TCCAGAACACCTCTCGTCCA	ACTGCTTCATCAGGTGTCAGA	79	60
*nfe2l2a* ^1^	NM_182889.1	TGGCCCTGAAGAATTTAACG	CCCGGTGAGAAGCTCTGTAG	138	60
*actb * ^2^	NM_181601.5	CTGTTCCAGCCATCCTTCTT	TGTTGGCATACAGGTCCTTAC	109	60

^1^ [46]; ^2^ [47].

## Data Availability

All data supporting the findings of this study are included within the article.

## Abbreviations

The following abbreviations are used in this manuscript:

PESO	*Passiflora edulis* seed oil
ROS	Reactive oxygen species
DMSO	Dimethyl sulfoxide
PBS	Phosphate-buffered saline
FET	Fish Embryo Acute Toxicity Test
NOAEL	No Observed Adverse Effect Level
LC_50_	Median lethal concentration
LCmin	Minimum lethal concentration
rpm	Revolutions per minute
*sod1*	Superoxide dismutase 1
*sod2*	Superoxide dismutase 2
*cat*	Catalase
*gpx1a*	Glutathione peroxidase 1a
*gsr*	Glutathione reductase
*nf2l2a*	Nuclear factor erythroid 2–related factor 2
*elfa*	Elongation factor 1-alpha
*gapdh*	Glyceraldehyde-3-phosphate dehydrogenase
